# Curcumin targets the TFEB-lysosome pathway for induction of autophagy

**DOI:** 10.18632/oncotarget.12318

**Published:** 2016-09-28

**Authors:** Jianbin Zhang, Jigang Wang, Jian Xu, Yuanqiang Lu, Jiukun Jiang, Liming Wang, Han-Ming Shen, Dajing Xia

**Affiliations:** ^1^ Clinical Research Institute, Zhejiang Provincial People's Hospital, Hangzhou, China; ^2^ Department of Physiology, Yong Loo Lin School of Medicine, National University of Singapore, Singapore; ^3^ School of Public Health, Zhejiang University, Institute of Immunology, Zhejiang University, Hangzhou, China; ^4^ First Affiliated Hospital, College of Medicine, Zhejiang University, Hangzhou, China; ^5^ NUS Graduate School for Integrative Sciences and Engineering, National University of Singapore, Singapore

**Keywords:** Curcumin, lysosome, mTOR, TFEB, autophagy

## Abstract

Curcumin is a hydrophobic polyphenol derived from the herb *Curcumalonga* and its wide spectrum of pharmacological activities has been widely studied. It has been reported that Curcumin can induce autophagy through inhibition of the Akt-mTOR pathway. However, the effect of Curcumin on lysosome remains largely elusive. In this study, we first found that Curcumin treatment enhances autophagic flux in both human colon cancer HCT116 cells and mouse embryonic fibroblasts (MEFs). Moreover, Curcumin treatment promotes lysosomal function, evidenced by the increased lysosomal acidification and enzyme activity. Second, Curcumin is capable of suppressing the mammalian target of rapamycin (mTOR). Interestingly, Curcumin fails to inhibit mTOR and to activate lysosomal function in *Tsc2^−/−^*MEFs with constitutive activation of mTOR, indicating that Curcumin-mediated lysosomal activation is achieved via suppression of mTOR. Third, Curcumin treatment activates transcription factor EB (TFEB), a key nuclear transcription factor in control of autophagy and lysosome biogenesis and function, based on the following observations: (i) Curcumin directly binds to TFEB, (ii) Curcumin promotes TFEB nuclear translocation; and (iii) Curcumin increases transcriptional activity of TFEB. Finally, inhibition of autophagy and lysosome leads to more cell death in Curcumin-treated HCT116 cells, suggesting that autophagy and lysosomal activation serves as a cell survival mechanism to protect against Curcumin-mediated cell death. Taken together, data from our study provide a novel insight into the regulatory mechanisms of Curcumin on autophagy and lysosome, which may facilitate the development of Curcumin as a potential cancer therapeutic agent.

## INTRODUCTION

Autophagy is an evolutionarily conserved self-digestive process via which cells adapt to nutrient starvation and other stress conditions [1, 2]. Autophagy plays important roles in many physiological processes such as development, innate immune defense, protein quality control, cell survival, and cell death, as well as in the pathogenesis of important diseases including cancer, neurodegenerative diseases, and metabolic disorders [3, 4]. During autophagy, portions of cytoplasmic materials, including macromolecules and organelles, are engulfed into specialized double-membrane structures, autophagosomes, which then fuse with lysosomes to form autolysosomes for degradation of their cargos and regeneration of nutrients [5]. Under normal growth conditions when nutrients are abundant, autophagy is kept at a basal level mainly for housekeeping purposes such as degradation of long-lived proteins and turnover of damaged cellular organelles. Under stress conditions such as nutrient starvation, autophagy is activated to provide cells with essential nutrients to keep cell survival. This induction is largely mediated via suppression of mammalian target of rapamycin (mTOR), the key negative regulator of autophagy that directly phosphorylates the unc-51 like autophagy activating kinase 1 (ULK1) [6].

Curcumin is a hydrophobic polyphenol derived from turmeric: the rhizome of the herb *Curcumalonga*. It is known that Curcumin is a highly pleiotropic molecule with abundant targets to exert its pharmacological functions [7]. The anti-cancer property of Curcumin has been well studied in a variety of cancers, such as breast cancer, prostate cancer and melanoma [8, 9]. At present, several studies have demonstrated that Curcumin is capable of inducing autophagy in many types of human cancer cells, including human oral cancer, [10] colon cancer, [11] glioblastoma, [12] lung adenocarcinoma [13] and uterine leiomyosarcoma [14]. Additionally, Curcumin can protect human vascular endothelial cell, mouse cardiomyocytes and rat kidney cells from oxidative stress damage through induction of autophagy [15–17].

At present, there are two major issues in Curcumin-mediated autophagy. First, it remains controversial whether such inducible autophagy serves as a pro-survival or pro-death mechanism. It has been reported that autophagy inhibition enhances Curcumin-caused cell death, indicting the pro-survival function of autophagy [18, 19]. In contrast, Curcumin has been shown to induce autopahgic cell death [20]. Second, the molecular mechanisms underlying Curcumin-induced autophagy have not been fully understood. There are several signaling pathways involved in different cancer cells, such as the downregulation of the PI3K-Akt-mTOR signaling pathway, [21–23] activation of AMP-activated protein kinase (AMPK) pathway [13] and ERK1/2 pathway, [14, 23] production of reactive oxygen species (ROS) [11] and occurrence of ER stress [24]. However, little is known whether Curcumin has any direct effect on lysosome.

Lysosomes are acidic organelles containing a wide spectrum of hydrolytic enzyme, including proteases, peptidases, phosphatases, nucleases, glycosidases, sulfatases, and lipases designated for all types of macromolecules [25]. In the course of autophagy, lysosome plays an essential role in the maturation/degradation stage of autophagy, as the contents in the autophagosomes are eventually degraded by lysosomes, via autophagosome-lysosome fusion [26]. The most important molecular mechanism in regulation of lysosomal function is the nuclear transcriptional factor EB (TFEB) which is the direct downstream target of mTOR [27–29]. Under normal condition, the active mTOR complex 1 (mTORC1) directly phosphorylates TFEB and suppresses its transcriptional activities by controlling its cytosolic localization. Under starvation condition or upon mTOR inhibition, dephosphorylated TFEB translocates to nuclei and promotes the transcription of its target genes that are known to play important roles in autophagy and lysosome activation.

It has been reported [30] that lysosomal membrane permeabilization is involved in Curcumin-induced apoptosis of lung carcinoma cells. More recently, a synthesized Curcumin derivative termed C1 has been shown to enhance autophagy and lysosome biogenesis via activation of TFEB in mouse neuroblastoma neuro-2a (N2a) cells and HeLa cells [31]. However, the effect of Curcumin itself on TFEB and lysosome remains largely unknown.

In this study, we aimed to examine the importance of TFEB and lysosome in Curcumin-induced autophagy in human cancer cells. Our study indicates that Curcumin directly binds to TFEB, increases TFEB transcriptional activity and activates lysosomal function. Moreover, induction of autophagy and lysosomal activation by Curcumin serves as a cell survival. Data from this study thus shed new lights into the anti-cancer potential of Curcumin.

## RESULTS

### Curcumin induces autophagy in HCT116 and MEF cells

**Figure 1:**
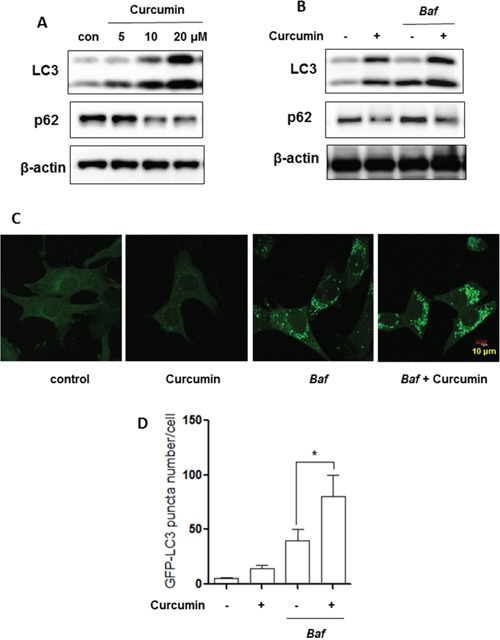
Curcumin induces autophagy **A.** HCT116 cells were treated with Curcumin (5, 10 or 20 μM) for 12 hours. **B.** HCT116 cells were treated with Curcumin (10 μM, 12 hrs) in the presence or absence of bafilomycin A1 (*Baf*, 15 nM). Cells were harvested for western blotting to determine LC3-II and p62 level. β-actin was used as a loading control. **C.** and **D.** Mouse embryonic fibroblasts (MEFs) with stable expression of GFP-LC3 were treated with Curcumin (20 μM, 12 hrs) with or without bafilomycin A1 (*Baf*, 15 nM) and GFP-LC3 puncta in treated cells was measured and quantified by confocal microscopy (Scale bar 10 μm). All values are means ± SD at least 3 independent experiments. Student's *t* test, * *P* < 0.05.

In this study we first examined the effect of Curcumin on autophagy. We found that Curcumin treatment increased LC3-II level in human colorectal cancer HCT116 cells in a dose-dependent manner (Figure 1A), a commonly used autophagy marker [32]. Meanwhile, the autophagy flux was also determined by p62 level or by using bafilomycin A1 (*Baf*, a vacuolar-type H^+^-ATPase inhibitor that blocks autophagosome and lysosome fusion) [33]. As shown in Figure 1A, Curcumin reduced the p62 level in a dosage-dependent manner. Moreover, Curcumin led to a further increase of LC3-II level in HCT116 cells in the presence of *Baf* (Figure 1B). Consistently, the number of GFP-LC3 puncta was further increased by Curcumin in the presence of *Baf* in MEFs with stable expression of GFP-LC3 (Figure 1C and 1D) [34]. All these results demonstrate the increased autophagic flux in cells treated with Curcumin.

### Activation of lysosomal function in Curcumin-treated cells

**Figure 2:**
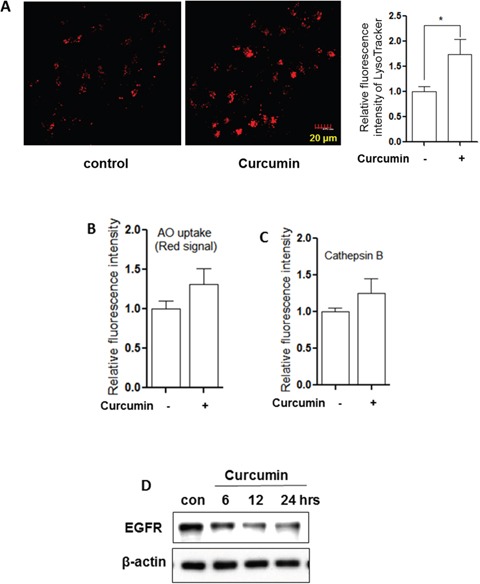
Curcumin activates lysosomal function **A.** HCT116 cells were treated with Curcumin (10 μM) at indicated concentration for 12 hours and then stained with LysoTracker Red DND-99 (50 nM) for 15 minutes. Fluorescence intensity of treated cells was measured by confocal microscopy (left) or flow cytometry (right). The numeric data are presented as means ± SD from 2 independent experiments. Student's *t*-test, * *P* < 0.05. **B.** and **C.** as indicated in A., after 12 hour treatment, AO staining and Magic Red (Cathepsin B) were performed and analysed using flow cytometry. **D.** HCT116 cells were treated with Curcumin (10 μM) for different time (6, 12 or 24 hrs) and EGFR was analysed using western blotting. β-actin was used as a loading control.

In order to examine the effect of Curcumin on lysosome, we used several assays to test the changes of lysosomal function in HCT116 cells. First, as shown in Figure 2A, LysoTracker staining showed that cell fluorescence intensity was increased by Curcumin in HCT116 cells, indicating enhanced acidification of lysosome (reduced pH). This was also confirmed by an increase of red signal using acridine orange (AO) staining (Figure 2B), an orange/red fluorescent chelating dye accumulation in acidic organelle lysosome [35]. Second, the enzyme activities of lysosomal Cathepsin B were also measured using Cathepsin Magic Red™. There was a 1.5-fold increase of cell fluorescence intensity after 12 hours Curcumin treatment in HCT116 cells (Figure 2C). Third, we measured changes of EGFR protein level, which is known to be mediated by lysosome degradation [36]. As shown in Figure 2D, a time-dependent degradation of EGFR was observed in HCT116 cells by Curcumin, suggesting the increased lysosomal degradative function.

### Lysosomal activation by Curcumin is due to mTOR suppression

**Figure 3:**
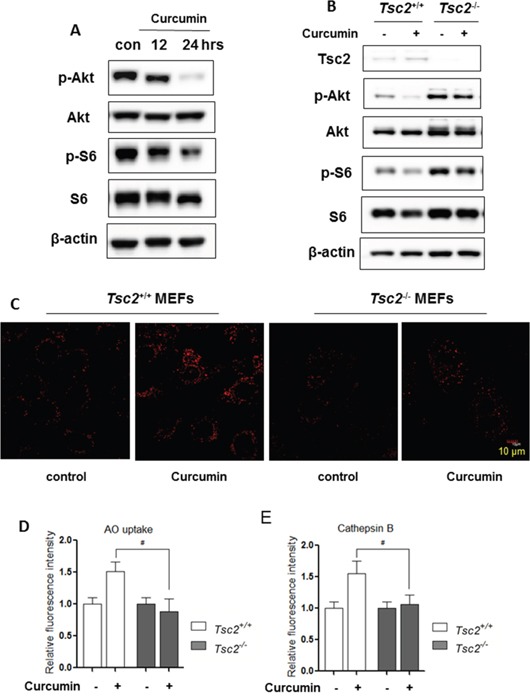
Activation of lysosomal function by Curcumin is mTOR-dependent **A.** HCT116 cells were treated with Curcumin (10 μM) as indicated. **B.** *Tsc2^+/+^* and *Tsc2^−/−^* MEFs were treated with Curcumin (20 μM) for 12 hours. Cells were harvested and lysed for western blotting to determine phospho-Akt (Ser473) and phospho-S6 (Ser235/236). β-actin was used as a loading control. **C.** as indicated in (B), LysoTracker Red staining was performed using confocal microscopy (Scale bar 10 μm) while MagicRed Cathepsin B **D.** and AO staining **E.** were also performed and were treated with Curcumin (20 μM) for 12 hours and analysed in *Tsc2^+/+^* and *Tsc2^−/−^* MEFs using flow cytometry. All values are means ± SD at least 3 independent experiments and analysed using Student's *t* test (*P < 0.05).

One of most important molecular mechanisms in regulation of lysosomal function in the course of autophagy is depending on mTOR inhibition [29, 37]. As shown in Figure 3A, Curcumin treatment reduced phospho-Akt and phospho-S6 level in HCT116 cells in a time-dependent manner, indicating the suppression of the Akt-mTOR pathway.

To further establish the role of mTOR in regulating lysosomal function, we utilized the *Tsc2^−/−^* MEFs in which the mTOR is constitutively active [38]. In Figure 3B, Curcumin treatment for 12 hours failed to suppress mTOR activity in the *Tsc2^−/−^* MEFs (no reduction of both phospho-Akt and phospho-S6). Meanwhile, in *Tsc2^−/−^* MEFs, LysoTracker staining (Figure 3C), AO staining (Figure 3D) and Magic Red Cathepsin B staining (Figure 3E) showed that Curcumin treatment failed to increase the cells' fluorescence intensity, suggesting that Curcumin is unable to induce lysosomal activation in *Tsc2^−/−^* MEFs. These results thus indicate lysosomal activation by Curcumin is most probably mediated via its suppressive effects on mTOR.

### Curcumin directly binds to TFEB and increases its transcriptional activity

**Figure 4:**
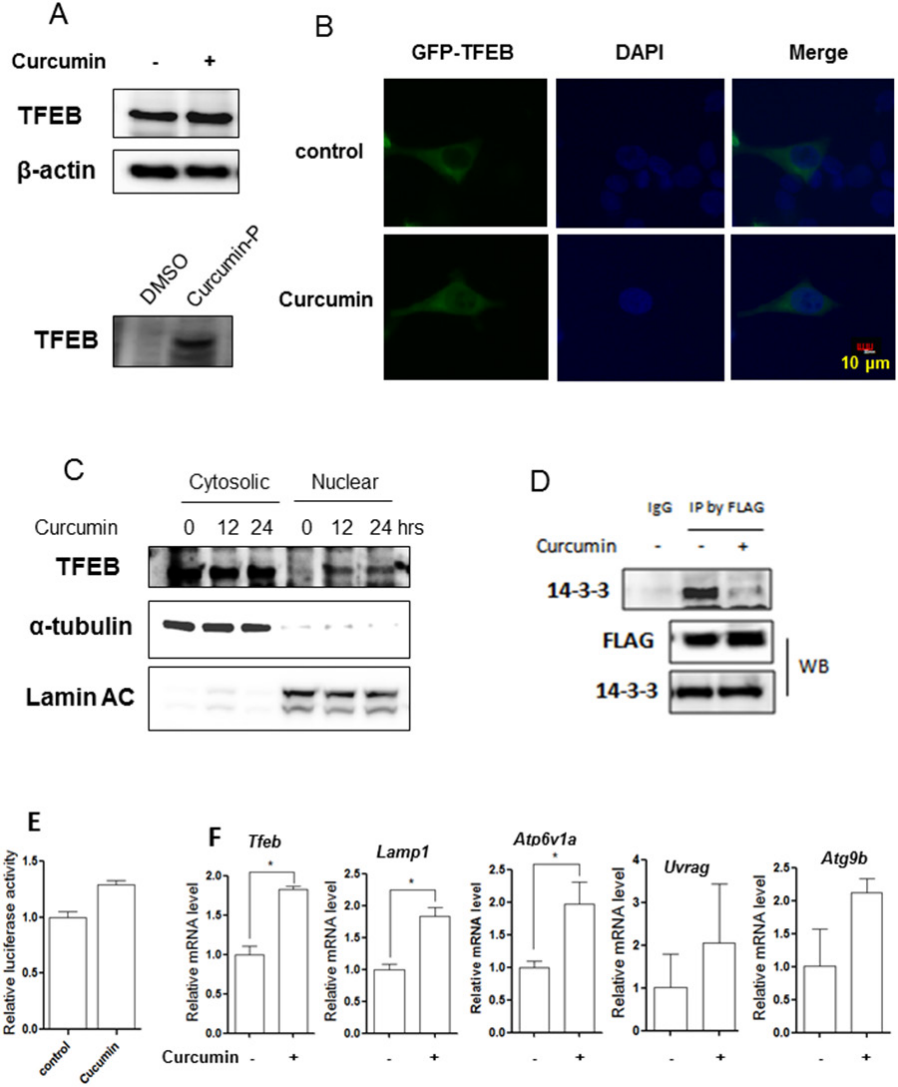
Curcumin directly targets TFEB for activation **A.** HCT116 cells were treated with 10 μM Curcumin for 12 hours and cell lysates were prepared followed by immunoblotting for TFEB and β-actin (up panel). HCT116 cells were labeled with Curcumin-probe (30 μM) for 4 hours and western blotting was performed to validate Curcumin-probe targeted TFEB (down panel). **B.** Enhanced TFEB nuclear translocation in response to Curcumin treatment (10 μM; 12 hours). Live imaging of GFP-TFEB (green) and DAPI (blue) in HCT116 cells showed an enrichment of the GFP-TFEB signal in the nuclear. Five fields containing 20 to 30 cells were analyzed for TFEB nuclear localization. Scale bar, 10 μm. **C.** HCT116 cells were treated with 10 μM Curcumin as indicated. Cytosolic and nuclear fraction from control and Curcumin-treated cells were probed for TFEB. The same membrane was then stripped and reprobed for α-tubulin or Lamin AC as loading control. **D.** HCT116 cells were transient transfected with the TFEB-3x Flag (kindly provided by Dr. A Ballabio) and then treated with 10 μM Curcumin for 12 hours. Cells were lysed and subjected to immunoprecipitation with anti-FLAG antibody followed by immunoblotting for 14-3-3. TFEB was also determined using anti-FLAG antibody. **E.** HCT116 cells were transiently transfected with the TFEB-luc reporter construct (kindly provided by Dr. A Ballabio). After 24 hours, the cells were treated with Curcumin (10 μM) for another 12 hours and the relative luciferase activity was measured. RLU refers to relative luciferase units. Error bars represent the standard deviation from two independent experiments. **F.** HCT116 cells were treated with Curcumin (10 μM) for 12 hours and cells were harvested for RNA extraction. Real-time PCR was performed to determine mRNA level changes in the known TFEB target genes, such as *Lamp1, Atp6v1a, Uvrag* and *Atg9b*. *Gapdh* was used as a loading control. All values are means ± SD at least 3 independent experiments. Student's *t* test, * *P* < 0.05.

It has been well established that TFEB is a master regulator for lysosomal biogenesis by driving expression of autophagy and lysosomal-related genes [28, 39]. Here, we treated HCT116 cells with Curcumin and found that Curcumin treatment did not increase TFEB protein level (Figure 4A). To validate whether TFEB serves as a direct molecular target of Curcumin, we used a synthesized cell permeable curcumin probe (Cur-P) with an alkyne moiety, [40] which can be tagged with biotin for affinity enrichment of the direct-binding protein targets of Curcumin in situ. HCT116 cells were first treated with a Curcumin-probe and then cell lysate was prepared to react with Rhodamine B-azide through click chemistry followed by SDS-PAGE. As shown in Figure 4A, Curcumin-probe directly binds to TFEB in HCT116 cells, suggesting that TFEB is one of Curcumin molecular targets.

Next, we measured the transcriptional activity of TFEB after Curcumin treatment, including its nuclear translocation and its transcriptional regulation of its target genes. First, the localization of TFEB was determined after Curcumin treatment. In HCT116 cells with transient expression of GFP-TFEB, Curcumin microscopy analysis showed that TFEB translocated into the nuclear after (Figure 4B). To further confirm its nuclear localization, we prepared cellular fractions from HCT116 cells and western blotting also showed nuclear translocation of TFEB after Curcumin treatment (Figure 4C). Moreover, we determined whether the nuclear translocation of TFEB by Curcumin is associated with its phosphorylation. Phosphorylation level in TFEB was indirectly investigated by measuring its interaction with protein 14-3-3, which is known that mTOR controls TFEB activity via regulation of the serine 211 phosphorylation-dependent binding of 14-3-3 proteins to TFEB [41]. As shown in Figure 4D, in cells with transient overexpression of FLAG-TFEB, treatment with Curcumin led to a significant decrease in the interaction of TFEB with 14-3-3, indicating the reduced phosphorylation level of TFEB.

To further confirm the changes of transcriptional activity of TFEB, a TFEB promoter driven luciferase reporter construct was transiently transfected into HCT116 cells, followed by Curcumin treatment for 12 hours. As shown in Figure 4E, the relative luciferase activity of TFEB was markedly increased by Curcumin. Finally, the mRNA level of some known TFEB target genes, such as the V-ATPase subunits, *Lamp1* and *Atg*s, were measured. In HCT116 cells, Curcumin treatment significantly upregulated some TFEB target genes expression, including *Tfeb, Lamp1, Atp6v1a, Uvrag, Atg9b* (Figure 4F). Our data thus suggest that Curcumin activates TFEB transcriptional activity.

### Lysosomal activation by Curcumin serves as a cell survival mechanism

**Figure 5:**
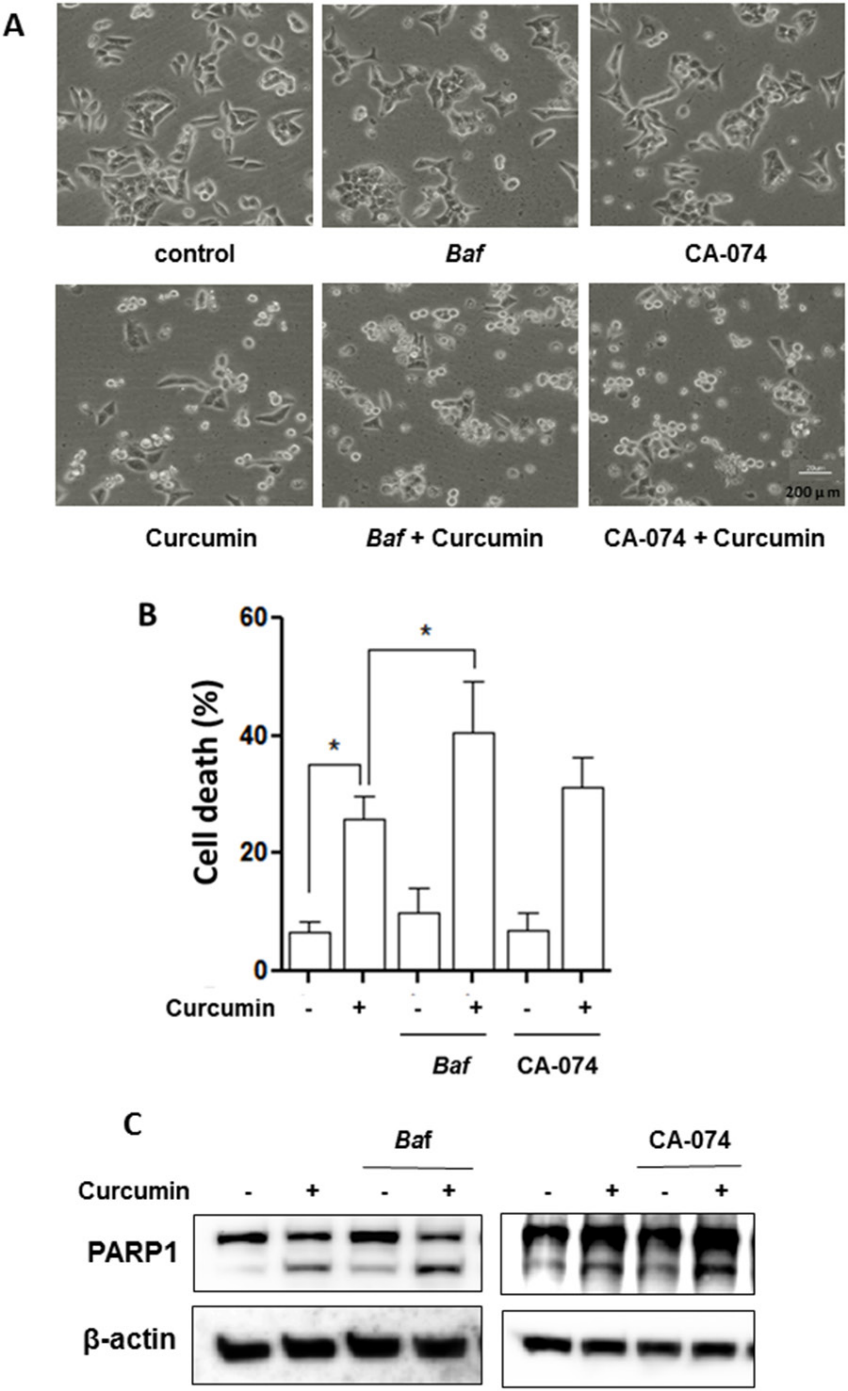
Lysosomal inhibition sensitizes curcumin-induced cell death HCT116 cells were treated with Curcumin (20 μM), with or without bafilomycin A1 (*Baf*, 25 nM) or CA-074 (25 μM) for 24 hours. **A.** Morphological changes of HCT116 cells with respective treatments were examined and photographed with an inverted microscope (Scale bar 200 μm). **B.** Cell pellets were subsequently collected and cell death was quantified using propidium iodide (PI) exclusion staining. Statistical significance (* *P* < 0.05) is indicated in the bar chart. **C.** Cells were then harvested for protein analysis. Cell lysates were resolved in SDS-PAGE and probed with specific antibody against PARP1.

At present, the role of autophagy induced by Curcumin remains controversial [20, 42]. In this part of our study we determined the functional role of lysosome activation in Curcumin-induced cell death. First, we used two pharmacological inhibitors of lysosome: *Baf*, an inhibitor of vacuolar type H^+^-ATPase and CA-074, an Cathepsin B inhibitor [43]. As shown in Figure 5A, the morphological changes showed that Curcumin caused more cell death in HCT116 cells in the presence of *Baf* or CA-074.

Next, we performed propidium iodide (PI) exclusion test coupled with flow cytometry to quantify cell death in Curcumin-treated HCT116 cells. As shown in Figure 5B, similar results were also found in both HCT116 cells treated with Curcumin in the presence of BAF or CA-074. Such observations thus indicate that Curcumin-caused lysosomal activation serves as cell survival.

Finally, we examined the format of the cell death via detection of one apoptotic marker, the cleavage of PARP1 (poly [ADP-ribose] polymerase1). As shown in Figure 5C, there was evident cleavage of PARP1 in Curcumin-treated HCT116 cells. Moreover, *Baf* or CA-074 also enhanced PARP1 cleavage by Curcumin, strongly indicating that lysosome inhibition is able to sensitize Curcumin-caused apoptotic cell death.

## DISCUSSION

In autophagic pathway, lysosome plays a key role by controlling both cellular clearance and energy production to respond to environmental cues [27, 39]. Curcumin, a natural compound in the spice turmeric, has been used for dietary and medicinal purposes for thousands of years in many parts of the World [7]. In this study, for the first time, we found that Curcumin enhances lysosomal function in both HCT116 cells and MEFs through direct targeting and activation of TFEB.

At present, there is accumulating evidence showing that Curcumin induces autophagy and such inducible autophagy in functionally implicated in its anticancer, antioxidant and anti-inflammatory properties [8, 42, 44]. Inhibition of the Akt-mTOR pathway and activation of ERK signalling have been studied as the main mechanisms in regulating Curcumin-mediated autophagy [45]. However, the role of lysosomal function in Curcumin-induced autophagy has still not been well determined. In both HCT116 cells and MEFs, we observed that Curcumin is capable of activating lysosomal function evidenced by (i) an increase of lysosomal acidification (Figure 2A, 2B, 3C and 3D), (ii) enhanced cathepsin B activity (Figure 2C and 3E); and (iii) increased EGFR degradation (Figure 2C). The above findings are consistent with a recent report showing that Curcumin analog C1, one of synthetic monocarbonyl analogs of Curcumin, promotes lysosomal biogenesis and degradation [31].

Data from this study demonstrate the underlying molecular mechanisms for lysosomal activation by Curcumin. First, mTOR suppression is required for Curcumin-induced lysosomal activation, based on two important findings: (i) Curcumin treatment inhibits the Akt-mTOR pathway (Figure 3A), which is consistent with the previous reports; [21, 46] (ii) Curcumin fails to block mTOR activity and increase lysosomal acidification and Cathepsin B activity in *Tsc2^−/−^* MEFs (Figure 3B-3E). Second, Curcumin directly binds to TFEB and increases TFEB transcriptional activity. At present, the exact nature of such binding is not clear and further experiments are needed to identify the Curcumin binding sites on TFEB. On the other hand, as a result of mTOR suppression, the phosphorylation level of TFEB via its interaction with protein 14-3-3 is reduced which leads to its nuclear translocation (Figure 4B-4C), its increased luciferase activity (Figure 4E) and its target genes' upregulation (Figure 4F), such as the V-ATPase subunits (*Atp6v1a*), *Lamp1, Uvrag, Atg9b*, which is an important molecular mechanism for lysosome biogenesis and function [47]. Our findings are generally consistent with a recent report in which Curcumin analog C1 enhances TFEB nuclear translocation and transcriptional activity [31]. Intriguingly, Curcumin analog C1 does not inhibit mTOR activity and its effect on TFEB transcriptional activity is mTOR-independent, [31]. One possibility for such a discrepancy is due to structural differences between Curcumin and its analog, and this analog may activate lysosomal function via another molecular mechanism different from Curcumin itself. In addition, it is still unknown how Curcumin suppresses mTOR activity. Xiao et al [13]. reported that Curcumin markedly increases the phosphorylation of AMPK and acetylCoA carboxylase. As upstream of mTOR signalling, activated AMPK by Curcuming may result in the mTOR suppression [48, 49]. More research is needed for fully understanding the mechanisms underlying the inhibitory effect of Curcumin on the PI3K-Akt-mTOR pathway.

In lung cancer cells, Wu et al [30]. reported that Curcumin induces lysosome-dependent cell death via lysosomal membrane permeabilization (LMP) and cytosolic relocation of Cathepsin B and Cathepsin D in A549 lung cancer cells. Moreover, inhibition of Cathepsin B by Z-FA-fmk was able to block Curcmin-induced cell apoptosis [30]. In contrast, our results showed that Curcumin activates lysosomal function instead of lysosomal damage in both HCT116 cells and MEFs. In addition, lysosome inhibition by *Baf* or CA-074 leads to more HCT116 cells death (Figure 5). Such observations indicate that the exact effects of Curcumin on lysosome could be context-dependent.

In summary, data from this study demonstrate that Curcumin induces autophagy, activates lysosomal function via its inhibitory effects on the Akt-mTOR signalling pathway and via direct targeting and activation of TFEB. These two mechanisms of action of curcumin cooperatively activate lysosomal function. Our findings thus reveal a novel insight into the regulatory mechanisms of Curcumin on autophagy and lysosome and provide novel insights into the potential anti-cancer function of Curcumin.

## MATERIALS AND METHODS

### Reagents and antibodies

The chemicals used in our experiments were: LysoTracker Red DND-99 (Invitrogen, L7528), Magic RedTM cathepsin B reagent with Acridine Orange (Immunochemistry Technologies, LLC, 937/6130), CA-074 (APExBIO, A1926), bafilomycin A1 (*Baf*, Sigma, B1793), acridine orange (AO) (Immunochemistry Technologies, LLC, 6130) and [3-(4,5-dimethylthiazol-2-yl)-5-(3-carboxymethoxyphenyl)-2-(4-sulfophenyl)-2H-tetrazolium, inner salt; MTS) (Promega, G5421). The Curcumin-probe (Curcumin-P) was readily synthesized by mono-alkylation of curcumin by propargyl bromide and its structure was verified by H-NMR, C-NMR and high resolution mass spectrometry [40].

The antibodies used in our experimentsincluded: microtubule-associated protein 1 light chain 3 (LC3) (Sigma, L7543), p62 (Sigma, P0067), Tsc2 (Cell Signaling Technology, 4308), phospho-S6(S235/236) (Cell Signaling Technology, 2211), S6 (Cell Signaling Technology, 2317), β-actin (Sigma, A5441), α-tubulin (Sigma, T6199), Lamin AC (Cell Signaling Technology, 2032), EGF receptor (Cell Signaling Technology, 4267), LAMP1 (Cell Signaling Technology, 9091), PARP1 (Cell Signaling Technology, 9542), TFEB (Bethyl Laboratories, A303-673A), FLAG (Sigma, F1804), 14-3-3 (Cell Signaling Technology, 9638), ANTI-FLAG® M2 Affinity Gel (Sigma, A2220).

### Cell culture

*Tsc2*^+/+^ and *Tsc2*^−/−^ MEFs were obtained from Dr. DJ Kwiatkowski (Brigham and Women's Hospital, Harvard University) (referred to as *Tsc2*^+/+^ and *Tsc2*^−/−^ MEFs hereafter). HCT116 cells were obtained from American Type Culture Collection (ATCC). All cell lines were maintained in DMEM (Sigma, D1152) containing 10% fetal bovine serum (HyClone, SV30160.03) in a 5% CO_2_ atmosphere at 37°C. All cell lines were maintained in DMEM (Sigma, D1152) containing 10% fetal bovine serum (Hy-Clone, SV30160.03) in a 5% CO_2_ atmosphere at 37°C.

### Estimation of intralysosomal pH using LysoTracker

The intralysosomal pH was estimated using LysoTracker, following manufacturer's instructions. The fluorescence intensity was observed under a confocal microscope (Olympus Fluoview FV1000) and representative cells were selected and photographed.

### Cathepsin B activity assay

Following an earlier report report, [50] cells were cultured in 12-well plates, after designated treatments, cells were further loaded with Magic Red Cathepsin B reagent for 15 minutes. Fluorescence intensities of 10, 000 cells per sample were measured by flow cytometry using the FACS cytometer (BD Biosciences).

### Acridine orange (AO) staining

Cell were stained with acridine orange (AO) at a concentration of 5 μg/ml for 15 minutes and then washed with PBS. AO is a lysosomotropic weak base and a concentration dependent meta-chromatic fluorophore. Fluorescence intensities of 10, 000 cells per sample were measured by flow cytometry using the FACS cytometer (BD Biosciences).

### Measurement of cellular fluorescence intensity using confocal microscopy

GFP-LC3-expressing stable MEFs were seeded to a coverglass slide chamber (Lab-Tek, NUNC, 155411). After the designated treatments, cells were examined and recorded using a confocal microscope (Olympus Fluoview FV1000) and representative cells were selected and photographed.

### Luciferase assay

TFEB luciferase vector was provided by Dr A Ballabio [51]. The transient transfection of luciferase vector was performed in HCT116 cells using Lipofectamine 2000 transfection reagent (Invitrogen, 11668) according to the manufacturer's protocols. After the designated treatments, the luciferase activity was measured 48 hours after transfection using a Dual-Luciferase Reporter Assay System (Promega, E1960) based on the protocol provides by the manufacturer.

### Cell fractions preparation

HCT116 cells were treated with Curcumin at different time points. After that, nuclear andcytosolic extracts were then prepared with NE-PER® nuclear and cytoplasmic extraction reagents (Pierce, 78833) according to the manufacturer's protocol.

### Plasmids and transient transfection

pCMV-TFEB-3X Flag plasmid was provided by Dr A Ballabio [28]. HEK293T cells were transfected with pCMV-TFEB-3x Flag plasmid using Lipofectmine™ 3000 (Invitrogen, L3000015) for 48 hours according to the manufacturer's protocol and then followed by the designated treatments.

### *In situ* labeling of Curcumin-probe

HCT116 cells were cultured in six-well plates until 80-90% confluence was reached. As described before, [40] Curcumin-probe (30 μM) in 2 ml of medium with a final DMSO concentration of 1% was added, and the cells were incubated at 37°C with 5% CO_2_ for 4 h. After treatment, the cells were pelleted, resuspended in PBS, washed, and subjected to sonication in 100 μl of PBS to lyse the cells. Centrifugation (10,000 rpm; 45 min) was applied to remove the insoluble fraction from the cell lysate. Equal amounts (50 μg) of the extracted proteins were then subjected to fluorescence labeling. The click reaction was done by adding Rhodamine B-azide (10 μM), TCEP (1 mM), TBTA (100 μM), and CuSO4 (1 mM) to the lysate, followed by 2 h-incubation at room temperature. The labeled proteins were then acetone-precipitated and air-dried. The samples were then solubilized with 100 μl of 1× SDS loading buffer.

### Immunoprecipitation (IP)

The immunoprecipitation assay was performed based on previously reports with minor modifications [52]. Briefly the cells were lysed on ice for 30 minutes with the IP buffer (40 mM HEPES, pH 7.4, 120 mM NaCl, 2 mM EDTA, 0.3% CHAPS, 10 mM pyrophosphate, 10 mM glycerophosphate, 50 mM NaF, phosphatase and protease inhibitor mixture). Cell lysates which containing 1 mg protein of each treatment were incubated with anti-FLAG® M2 Affinity Gel and mixed overnight with gentle rocking at 4°C. Then, immunoprecipitates were washed 3 times in IP Buffer and the immunoprecipitated complexes were eluted by boiling for 5 minutes in sample buffer (Bio-Rad). Lastly, the eluted immunoprecipitated complexes were resolved on SDS-PAGE gel and transferred onto PVDF membrane (Bio-Rad) for immunoblotting analysis.

### Immunoblotting

At the end of the designated treatments, cells were lysed in Laemmli SDS buffer (62.5 mM Tris, pH 6.8, 25% glycerol, 2% SDS, phosphatase inhibitor and proteinase inhibitor cocktail). An equal amount of protein was resolved by SDS-PAGE and transferred onto PVDF membrane. After blocking with 5% nonfat milk, the membrane was probed with designated primary and secondary antibodies, developed with the enhanced chemiluminescence method and visualized withthe ImageQuant LAS 500 (GE Healthcare).

### Reverse transcription and quantitative real-time PCR

RNA was extracted with the RNeasy kit (Qiagen, 217004). A reverse transcription reaction was performed using 1 μg of total RNA with High Capacity cDNA Reverse Transcription kit (Applied Biosystems, 4368814). The mRNA expression levels were determined by real-time PCR using SsoFast EvaGreen Supermix (Bio-Rad, 172-5201) and CFX96 Touch Real-time PCR Detection System (Bio-Rad). Glyceraldehyde-3-phosphate dehydrogenase (Gapdh) was used as an internal control of RNA integrity. Real-time PCR was performed in triplicate. The primers used for *Atp6v1a, Atg9b, Lamp1, Tfeb, Uvrag* and *Gapdh* were based on the previous report [47].

### Detection of viable and dead cells

Three assays were used to detect cell death quantitatively and qualitatively, which are (i) morphological changes under phase-contrast microscopy, (ii) propidium iodide (PI) live cell uptake assay coupled with flow cytometry and (iii) western blotting for PARP1 cleavage. For PI staining, the medium in each well was collected and cells were harvested with trypsin after treatments. Then, cell pellets obtained were resuspended in 1× phosphate-buffered saline (1st Base, Singapore, BUF-2041) containing PI at a final concentration of 5 μg/ml and incubated for 10 minutes at 37°C. Ten thousand cells from each sample were analyzed with FACS Calibur flow cytometry (BD Bioscience, San Jose, CA) using CellQuest software.

### Statistical analysis

All western blot and image data presented are representatives from at least three independent experiments. The numeric data are presented as means ± SD from 2-3 independent experiments (each in duplicates or triplicates) and analyzed using Student's *t*-test.

## Abbreviations

BAF: bafilomycin A1
CTSB: Cathepsin B
EGFR: epidermal growth factor receptor
LAMP1: lysosomal-associated membrane protein 1
mTOR: mammalian target of rapamycin
MAP1LC3/LC3: microtubule-associated protein1 light chain 3
TFEB: transcription factor EB
